# Structural and Functional Assessment of the Macular Inner Retinal Layers in Multiple Sclerosis Eyes Without History of Optic Neuropathy

**DOI:** 10.3390/jcm14165919

**Published:** 2025-08-21

**Authors:** Lucilla Barbano, Lucia Ziccardi, Carmen Dell’Aquila, Mattia D’Andrea, Carolina Gabri Nicoletti, Doriana Landi, Giorgia Mataluni, Antonio Di Renzo, Fabio Buttari, Roberto dell’Omo, Girolama Alessandra Marfia, Diego Centonze, Vincenzo Parisi

**Affiliations:** 1 IRCCS–Fondazione Bietti, Via Livenza 1, 00198 Rome, Italy; lucilla.barbano@fondazionebietti.it (L.B.); carmen.dellaquila@fondazionebietti.it (C.D.); antonio.direnzo@fondazionebietti.it (A.D.R.); vincenzo.parisi@fondazionebietti.it (V.P.); 2 Department of Medicine and Health Sciences “Vincenzo Tiberio”, University of Molise, Via Francesco De Sanctis 1, 86100 Campobasso, Italy; roberto.dellomo@unimol.it; 3 Department of Sense Organs, Faculty of Medicine and Dentistry, Sapienza University of Rome, Viale del Policlinico 155, 00161 Rome, Italy; mattia.dandrea@uniroma1.it; 4 Multiple Sclerosis Unit, Policlinico Tor Vergata, Viale Oxford 81, 00133 Rome, Italy; carolgabri@gmail.com (C.G.N.); doriana.landi@gmail.com (D.L.); giorgia.mataluni@gmail.com (G.M.); marfia@uniroma2.it (G.A.M.); centonze@uniroma2.it (D.C.); 5 Department of Systems Medicine, Tor Vergata University, Via Montpellier 1, 00133 Rome, Italy; fabio.buttari@gmail.com; 6 Unit of Neurology, IRCCS–Neuromed, Via Atinense 18, 86077 Pozzilli, IS, Italy

**Keywords:** multifocal photopic negative response, inner retina, multiple sclerosis, macular function, OCT

## Abstract

**Background**: Considering the lack of studies regarding the localized evaluation of the macular inner retina in multiple sclerosis patients without optic neuritis (MSnoON eyes), we investigated the structure and function of retinal ganglion cells (RGCs) located in different macular areas. **Methods**: In 24 MSnoON patients (mean age: 45.22 ± 5.57 years; 14 females and 10 males; mean MS disease duration: 11.07 ± 5.88 years) and in 30 age-similar (mean age: 45.09 ± 5.08 years) control subjects, complete ophthalmological examination, optical coherence tomography (OCT) and multifocal photopic negative response (mfPhNR) were performed. The ganglion cell layer thickness (GCL+-T) via OCT and the response amplitude density (RAD) through mfPhNR were measured from localized macular regions, including rings and Early Treatment of Diabetic Retinopathy Study (ETDRS) sectors. **Results**: When comparing MSnoON data from all tested areas with respect to the controls, macular GCL+-T and mfPhNR RAD mean values were found to be significantly (ANOVA, *p* < 0.01) reduced. In the MSonON group, considering both rings and sectors, the GCL+-T values were significantly and linearly correlated (Pearson’s test, *p* < 0.01) to the mfPhNR RAD values. **Conclusions**: In MS, even in the absence of optic neuritis, potential primary morpho-functional involvement of the inner macular elements can occur. This impairment widely involves all macular areas and sectors.

## 1. Introduction

Visual impairment in multiple sclerosis (MS), a chronic demyelinating disorder of the central nervous system, is characterized by either visual or motor dysfunction [1]. Axonal impairment and neuronal degeneration may be secondary to both anterograde and retrograde injury, even in early stages of the disease [1].

The involvement of the retina, and particularly of the macular unmyelinated axons of the retinal ganglion cells (RGCs), has received great attention in the last few years [2,3,4,5]. These neuronal elements, in fact, can be considered as the cellular target of the retinal neurodegenerative process due to MS, as a consequence of the frequent inflammatory process of the optic nerve, known as optic neuritis (ON) [2,3,4,5,6].

The reliable in vivo structural evaluation of macular layers by means of optical coherence tomography (OCT) has identified reduced thickness of the complex made of the ganglion cell/inner plexiform layer (GCIPL), providing evidence of early inner retina impairment in MS after ON (MSON eyes) [3,4,5,7,8,9].

On the functional side, the reduced amplitude of the pattern electroretinogram (PERG), originating from central RGCs and relative fibers, has been recognized as a valuable hallmark for the functional impairment of ganglionic retinal elements and their axons in MSON eyes [10,11,12].

More recently, the novel technique of the multifocal photopic negative response (mfPhNR) has been applied for studying macular inner retinal function using specific topographies [13,14]. The unique nature of this technical procedure is the possibility of comparing functional results (mfPhNR) with morphological changes (assessed by OCT) in corresponding and almost-overlapping macular areas [14]. In MSON eyes, for the first time, our group [14] described reduced mfPhNR signals by studying RGC structure and function in localized concentric rings and sectors, reproducing the Early Treatment of Diabetic Retinopathy Study (ETDRS) map and describing that inner retinal neurodegeneration involves foveal and parafoveal areas, likely reflecting a widespread phenomenon after an episode of ON.

In eyes with MS but without a history of ON (MSnoON eyes), although previous OCT segmentation analyses of the inner retina have documented reductions in the macular retinal nerve fiber layer (RNFL) and CGPIL thicknesses [2,3,4,15], there are few data on the functional condition of the macula. Indeed, the reported reduction in PERG amplitude in MSnoON has been considered a sign of inner retinal dysfunction, indicating that neurodegeneration of the visual system is not necessarily linked to the occurrence of ON [10,11,12]. Specifically, since only Wang et al. [16] studied the function of inner retina elements in MS patients (with and without ON) by recording the full-field photopic negative response (ff-PhNR) [17], reflecting the activity of whole RGCs and their axons, there is a lack of data on localized macular inner retinal dysfunction assessed through mfPhNR recordings.

Considering the lack of studies regarding the localized evaluation of the macular inner retina elements in MSnoON eyes, we aimed to assess the structure and function of the inner retina ganglionic elements in selected macular areas according to the topographical evaluation method previously applied to MSON eyes [14]. We compared MSnoON data with those of the same control group as in our previous published paper [14] to consistently evaluate a population of MS eyes with different characteristics, for instance, without a history of optic neuritis. In addition, our purpose was to observe whether a possible correlated macular morpho-functional involvement could be found in MSnoON eyes.

## 2. Materials and Methods

### 2.1. Study Design and Participants

Upon recruitment, each subject enrolled in this study signed informed consent. All procedures performed in this study adhered to the tenets of Declaration of Helsinki. The local Ethical Committee (Comitato Etico Territoriale Lazio Area 5, IRCCS Istituti Fisioterapici Ospitalieri, Roma, Italy) approved the study protocol (N.123/FB/24) on 15 April 2024.

One hundred relapsing-remitting MS patients were enrolled from trained neuro-ophthalmologists (L.B., L.Z., C.D., M.D.A., V.P.) at the Clinical and Research Center of Neurophthalmology and Rare Disease Unit of IRCCS-Fondazione Bietti. The patients were referred by expert neurologists in the field of multiple sclerosis (C.G.N., D.L., G.M., F.B., G.A.M., D.C.) from Tor Vergata University Hospital in Rome, between September 2024 and March 2025. From this MS cohort, we selected 24 MS patients (mean age 45.22 ± 5.57 years; 14 females and 10 males; average disease duration of 11.07 ± 5.88 years, range 5–20 years; mean Expanded Disability Status Scale score of 1.57 ± 1.59, range 0–3) without a history of ON in both eyes. The absence of ON was established based on the following: high-contrast best-corrected visual acuity (BCVA) of 0.0 LogMAR, absence of relative afferent pupillary reflex, absence of visual field defects detected by visual field (Humprey Field Analyzer SITA standard 30-2 strategy), normal color vision tested by Ishihara pseudo isochromatic plates, absence of ocular pain during eye movements, absence of signal abnormalities at the level of optic nerve on Magnetic Resonance Imaging, absence of abnormal delay of P100 implicit time and/or of abnormal reduction in N75-P100 amplitude at Visual Evoked Potentials recorded, according to ISCEV Standards [18]. 

From each MSnoON patient selected, only the left eye was examined, and, therefore, the MSnoON group provided 24 left eyes. The inclusion criteria were as follows: relapsing remitting MS in agreement with McDonald criteria [19] without a history of optic neuritis; age between 30 and 55 years; males or females; MS duration between 5 and 20 years (from the onset of symptoms to the last neurological visit); Expanded Disability Status Scale [20] between 0 and 3 assessed by two trained [21] neurologists (D.L. and G.M.); treatment with disease-modifying MS therapies [22]; and absence of other systemic diseases.

From the ophthalmological side, we selected MSnoOn with high-contrast BCVA of 0.0 LogMAR measured by ETDRS charts, with a mean refractive error of ±3.00 diopters spherical equivalent; normal intraocular pressure, absence of relevant ophthalmological diseases; absence of conditions that could interfere with a stable fixation during the instrumental examinations.

Data observed in the present MSnoON group were compared to control group, whose values were reported in our previously published work [14], to consistently describe macular RGC morpho-functional changes in MS eyes not affected by ON. Indeed, we aimed to compare the present MSnoON cohort of eyes with the same homogenous control group (30 age-similar subjects with mean age of 45.09 ± 5.08 years, 18 females and 12 males, and with 0.0 LogMAR BCVA) used for reported MSON eyes [14], to derive correct considerations on the morpho-function of the inner layers of the macula.

### 2.2. Optical Coherence Tomography

The automatic segmentation of the macular inner layers was performed using swept-source optical coherence tomography (Topcon DRI OCT Triton, Topcon, Japan) that measured the GCL+ as the sum of thickness of the following layers: macular retinal nerve fiber layer (RNFL), ganglion cell layer (GCL), inner plexiform layer (IPL) and inner nuclear layer (INL). The OCT examination was performed in mydriasis (Tropicamide 1%). Segmented layers were reviewed by two expert independent graders (L.B., L.Z.). APOSTEL recommendations were followed for quality control [23]. OCT image was considered only when the signal strength index was >50. Segmented inner macular layers to obtain the averaged GCL+ thickness (GCL+-T) measurements of controls and MSnoON eyes were measured via the macula 3D scan protocol.

**Figure 1:**
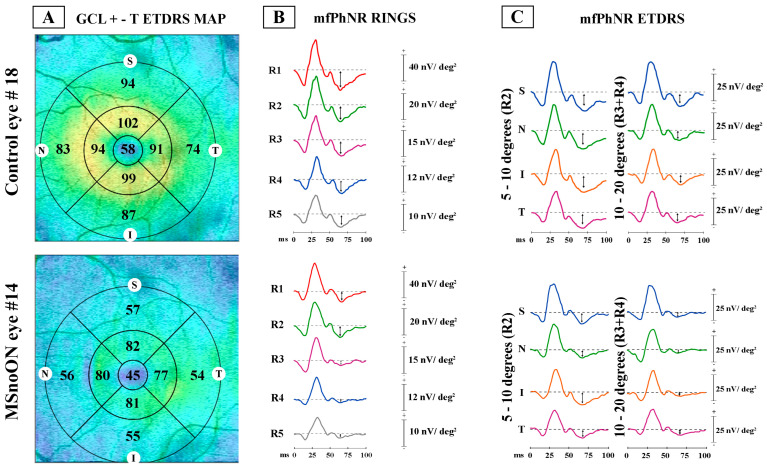
Examples of ganglion cell layer+-thickness (GCL+-T) and multifocal photopic negative response (mfPhNR) responses observed in a representative control eye (#18) and in a multiple sclerosis eye without a history of optic neuritis (#14). (**A**) GCL+-T data obtained in the ETDRS map configuration: GCL+-T value was measured in micron. Superior, S; nasal, N; inferior, I; and temporal, T sectors were considered. (**B**) MfPhNR response amplitude density [indicated by an arrow (↕) and measured in nanoV/degree^2^ (nV/deg^2^)] was calculated as baseline to trough within an implicit time between 50 and 90 milliseconds (ms). It represented the ring analysis of averaged traces obtained from 5 rings within 0–25° (R1, R2, R3, R4 and R5). (**C**) MfPhNR response amplitude density, in the configuration of Early Treatment Diabetic Retinopathy Study (ETDRS) analysis obtained from 4 sectors [superior (S), nasal (N), inferior (I) and temporal (T)] within 5–10° (R2) and within 10–20° (R3 + R4).

Figure 1 shows the analyzed ring and ETDRS topographies:

(1) Ring analysis: Area 1 encloses the 0–1 mm central ring, Area 2 encloses the 1–3 mm annular area (a mean value of 4 averaged sectors), and Area 3 encloses the 3–6 mm annular area (a mean value of 4 averaged sectors)
(2) ETDRS sector analysis: The GCL+-T was measured from the subfields within 1, 3, and 6 mm, respectively, defined by the ETDRS map. By considering ETDRS sectors between 1 and 3 mm (Area 2) and between 3 and 6 mm (Area 3), we obtained the averaged values of the superior (sup), nasal (nas), inferior (inf) and temporal (temp) sectors, each considered distinctly [23,24].

### 2.3. Multifocal Photopic Negative Responses Recordings

As reported in our previously published studies [14], we recorded the multifocal photopic negative response using an Espion system (Diagnosys UK, Ltd.; Histon, Cambridge, UK). The stimulus, projected on a monitor screen (stimulus frequency of 7 Hz, mean background luminance of 200 cd/m^2^, viewing distance of 33 cm) consisted of a circular stimulus of 60 elongated scaled dart pattern “segments”, interchanged between black (0 cd/m^2^) and white (400 cd/m^2^) according to an m-sequence of 12 bits. A central cross on the screen was used as target, for fixation purposes; meanwhile, the eye’s position was monitored on the screen of the computer, captured by a video system. Mydriasis up to a pupil diameter of 8 mm was required. The cornea was anesthetized for electrode setting (Benoxinate 0.4% eye drops).

To obtain the mfPhNR responses, binocular recordings were taken using active Dawson Trick Litzkow (DTL) bipolar contact electrodes and reference electrodes (Ag/AgCl skin electrodes placed on the corresponding outer canthi), with ground electrode (Ag/AgCl skin) placed in center of the forehead. Only first-order kernel responses from signals with inter electrode resistance lower than 3 KOhms, filtered (band pass 3–100 Hz) and after removal of artifacts, were considered.

The obtained mfPhNR traces were analyzed computing the average response amplitude densities (RADs) from different areas, measured in nanoVolt/degree^2^ (nV/deg^2^), as showed in Figure 1.

The mfPhNR RADs were analyzed in the following ways:

(1) Ring analysis, as proposed in previous reports for mfERG responses [25,26,27,28]. The responses derived from five annular rings (R) centered on the fovea: Ring 1 (R1) enclosing a 5° radius circular area, Ring 2 (R2) as an annular area between 5 and 10°, Ring 3 (R3) as an annular area enclosed between 10 and 15°, Ring 4 (R4) as the more external annular area between 15 and 20°, and Ring 5 (R5) as the outermost annular area between 20 and 25° (Ring 5, R5).
(2) ETDRS sector analysis, as previously evaluated by following the ETDRS OCT map [23,24]. We identified 9 sectors, where the central one corresponded to the R1 of the ring analysis (0–5°). Other sectors were the superior (sup), nasal (nas), inferior (inf) and temporal (temp) areas within 5–10° (R2). The outermost sectors analyzed the sup, nas, temp and inf areas within 10–20° (R3 + R4).

### 2.4. Statistical Analysis

The Gaussian distribution of our data was assumed by using the Kolmogorov–Smirnov test.

The sample size estimates were obtained by considering mfPhNR R1 RAD values from pilot evaluations (10 eyes from 10 MSnoON eyes and 10 eyes from 10 control subjects other than those included in the current study from unpublished data). The sizing was based on mfPhNR R1 RAD value of 26.27 ± 7.27 nV/deg^2^ for controls and 16.9 ± 7.9 nV/deg^2^ for MSnoON eyes [α = 5% (type 1 error) and power = 80% (β = 20%)] giving 12 participants for each group. We measured averaged values and standard deviation (SD) of structural and electrophysiological data from controls and MSnoON eyes and applied ANOVA to compare groups.

We performed a 1-sample *t*-test to calculate two-tailed 95% lower and upper confidence limits (CLs) for each parameter (GCL+-T and mfPhNR RAD values). Further, 95% CLs were obtained from control data.

To be consistent and to derive adequate conclusions, control group was the same as used in our previous study [14], evaluating the same inner macular morphological and functional parameters in MSON eyes.

Lastly, Pearson’s test was applied to correlate OCT values with the corresponding mfPhNR data in MSnoON eyes. A *p*-value < 0.01 was considered as statistically significant. SPSS (version 25) and Python 3.x (https://www.python.org) were used for statistical analysis.

## 3. Results

Table S1 reports the individual data of GCL+-T and mfPhNR RAD in MSnoON eyes observed in corresponding macular areas/rings or ETDRS sectors.

In Figure 1, representative examples of both morphological (GCL+ thickness assessed by OCT) and electrofunctional (mfPhNR recordings) analyses are presented, detected in localized retinal areas (rings and ETDRS sectors) in one control eye (#18) and in one eye with MSnoON (#14).

Table 1 shows the mean and SD values of GCL+-T detected in rings and ETDRS sectors (Area 1, Area 2, Area 3, Area 2 sup, Area 2 nas, Area 2 temp, Area 2 inf, Area 3 sup, Area 3 nas, Area 3 temp, Area 3 inf) and of mfPhNR RAD obtained by almost superimposed rings and ETDRS sectors (R1, R2, R3, R4, R5, R2 sup, R2 nas, R2 temp, R2 inf and R3 + R4 sup, R3 + R4 nas, R3 + R4 temp, R3 + R4 inf) in control and MSnoON groups. ANOVA statistical analyses between groups are also reported.

### 3.1. Ring Analysis of RGC Morphological Data

The individual macular GCL+-T values were reduced in 16/24 (66.66%) MSnoON eyes in Area 1 and in 19/24 (79.17%) and in 17/24 (70.83%) in Area 2 and in Area 3, respectively, considering the 95% lower confidence limit reported in Table S1.

As shown in Table 1, significantly (*p* < 0.01) reduced averaged GCL+-T values in Area 1, Area 2 and Area 3 were found in MSnoON with respect to the control group.

### 3.2. ETDRS Sector Analysis of RGC Morphological Data

The individual GCL+-T data from Area 2 were reduced in 19/24 (79.17%) MSnoON eyes in the superior and inferior sectors, in 18/24 (75.00%) eyes in the nasal sector and in 21/24 (87.50%) eyes in the temporal sector, considering the 95% lower confidence limit reported in Table S1,

On average, the GCL+-T values detected in the MSnoON group were significantly (*p* < 0.01) reduced in all ETDRS sectors when compared to the control group (see Table 1).

In Area 3, in MSnoON eyes, individual GCL+-T values were reduced in 14/24 (58.33%) eyes in the superior sector, in 16/24 (66.66%) eyes in the nasal sector, in 19/24 (79.17%) eyes in the inferior sector and in 15/24 (62.50%) eyes in the temporal sector.

On average (as reported on Table 1), in the MSnoON group, significantly (*p* < 0.01) reduced mean GCL+-T values were found in all ETDRS sectors with respect to the controls.

### 3.3. Ring Analysis of RGC Functional Data

The individual mfPhNR RAD values were reduced in 20/24 (83.33%) eyes in R1, in 19/24 (79.17%) eyes in R2 and R5 and in 18/24 (75.00%) eyes in R3 and R4. On average, in all rings, the RAD values detected in MSnoON groups were statistically significantly reduced (*p* < 0.01) compared to those observed in the control group (see Table 1).

### 3.4. ETDRS Sector Analysis of RGC Functional Data

In ETDRS R2 sectors, the individual mfPhNR RAD values were reduced in 20/24 (83.33%) eyes in the temporal sector, in 18/24 (75.00%) eyes in the superior sector and in 19/24 (79.17%) eyes in the nasal and inferior sectors.

On average, the mfPhNR RAD values detected in the MSnoON group were statistically significantly (*p* < 0.01) reduced in all ETDRS sectors when compared to control ones (see Table 1).

In ETDRS R3 + R4 sectors, in MSnoON eyes, reduced mfPhNR RADs were observed in 20/24 (83.33%) eyes in nasal and inferior sectors and in 19/24 (79.17%) eyes in superior and temporal sectors.

Table 1 shows significantly (*p* < 0.01) reduced averaged values of the mfPhNR RADs in the MSnoON group, as compared to the control group for all analyzed sectors.

### 3.5. Correlations Between Structural and Functional RGC Changes in MSnoON Eyes

Considering Table S1 and Table 2, in MSnoON eyes, a concomitant reduction in GCL+-T and of mfPhNR RAD values was detected in the greatest percentage of eyes, ranging from 62.50% in R1 and Area 1 to 83.33% of ETDRS Area 2 and ETDRS R2 temporal.

In MSnoON eyes, the concomitant presence of both normal mfPhNR RADs and GCL+ thickness from 3/24 (12.50%) eyes to 5/24 (20.83%) was found.

In several MSnoON eyes (ranging from 0% to 20.83%), reduced mfPhNR values but normal GCL+ thickness values were detected; by contrast, normal mfPhNR values and reduced GCL+ thickness were not observed in any of the MSnoON eyes.

**Figure 2:**
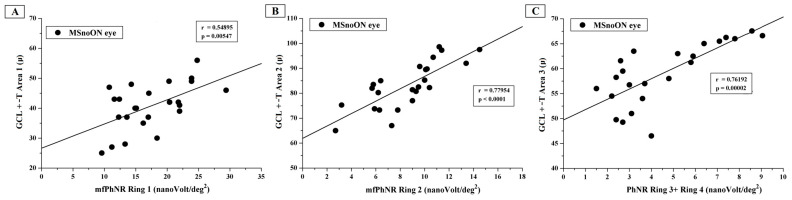
(**A**) The ganglion cell layer complex thickness (GCL+-T) measured in Area 1 plotted as a function of multifocal photopic negative response (mfPhNR) average response amplitude densities (RADs) recorded from Ring 1. (**B**) The GCL+-T values from Area 2 plotted as a function of the mfPhNR RAD recorded from Ring 2. (**C**) The GCL+-T values from Area 3 plotted as a function of the mfPhNR RAD recorded from Ring 3 + Ring 4.

In particular, Figure 2 presents the individual values of GCL+-T observed in Area 1, Area 2 and Area 3 plotted as a function of mfPhNR RAD values from R1, R2 and R3 + R4, respectively.

**Figure 3:**
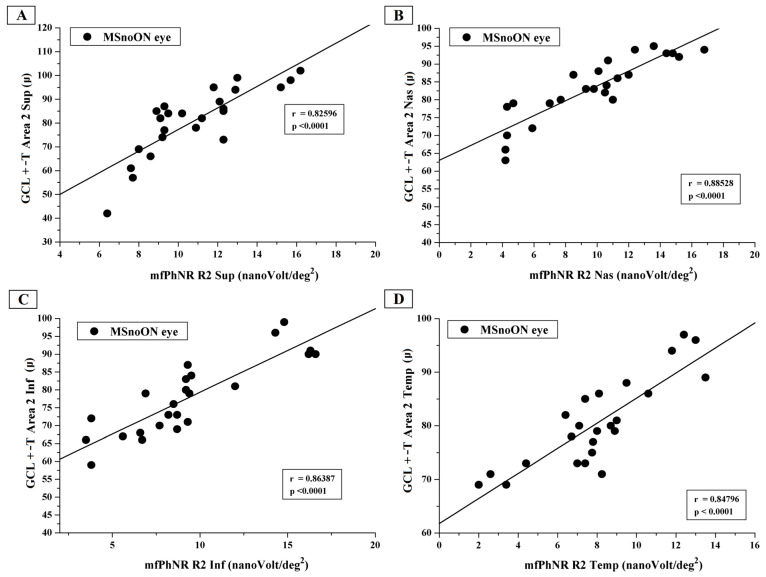
(**A**) The ganglion cells layer complex thickness (GCL+-T) measured in Area 2 superior plotted as a function of the multifocal photopic negative response (mfPhNR) average response amplitude densities (RADs) recorded in Ring 2 superior (R2 sup). (**B**) The GCL+-T measured in Area 2 nasal plotted as a function of the mfPhNR RAD recorded in Ring 2 nasal (R2 nas). (**C**) The GCL+-T measured in Area 2 inferior plotted as a function of the mfPhNRRAD recorded in Ring 2 inferior (R2 inf). (**D**) The GCL+-T measured in Area 2 temporal plotted as a function of the mfPhNR RAD recorded in Ring 2 temporal (R2 temp).

Figure 3 presents the individual values of GCL+-T from Area 2 sup, Area 2 nas, Area 2 inf and Area 2 temp plotted as a function of the mfPhNR RAD values from R2 ETDRS sectors (R2 sup, R2 nas, R2 inf and R2 temp).

**Figure 4:**
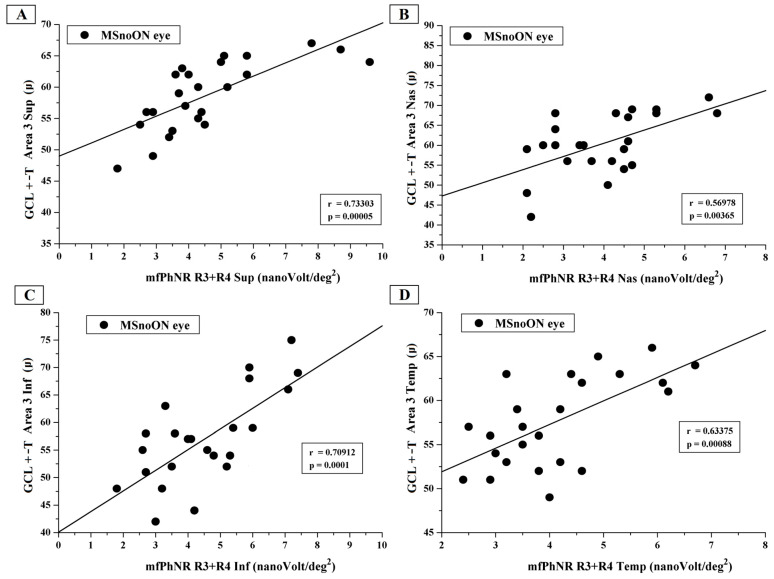
(**A**) The ganglion cell layer complex thickness (GCL+-T) measured in Area 3 superior plotted as a function of the multifocal photopic negative response (mfPhNR) average response amplitude densities (RADs) recorded in the Ring 3 + Ring 4 superior (R3 + R4 sup). (**B**) The GCL+-T measured in Area 3 nasal plotted as a function of the mfPhNR averaged RADs recorded in the Ring 3 + Ring 4 nasal (R3 + R4 nas). (**C**) The GCL+-T measured in Area 3 inferior plotted as a function of the mfPhNR averaged RADs recorded in the Ring 3 + Ring 4 inferior (R3 + R4 inf). (**D**) The GCL+-T measured in Area 3 temporal plotted as a function of the mfPhNR averaged RADs recorded in the Ring 3 + Ring 4 temporal (R3 + R4 temp).

Figure 4 shows the individual values of GCL+-T observed in Area 3 sup, Area 3 nas, Area 3 inf and Area 3 temp plotted as a function of the mfPhNR RAD values from R3 + R4 ETDRS sectors (R3 + R4 sup, R3 + R4 nas, R3 + R4 inf and R3 + R4 temp).

## 4. Discussion

In MSnoON eyes, we assessed the structure (via OCT) and the function (via mfPhNR) of the RGCs in selected macular areas. In addition, our purpose was to detect whether a possible correlated macular morpho-functional involvement of the inner retinal macular may occur.

### 4.1. Morphological Findings

As previously studied in different cohorts of MS eyes [2,3,14,29,30,31], and also in the present MSnoON cohort, we considered the OCT segmented macular RNFL-GCL-IPL measurement, called GCL+ thickness, as an indicator of inner retina structural integrity.

In our MSnoON cohort, we observed a statistically significant mean GCL+ thickness reduction in a range from 66.6% to 79.17% eyes in the examined circular areas (Area 1, Area 2, Area 3) and in a range from 58.33% to 87.50% eyes in the studied ETDRS sectors (Area 2 superior, Area 2 nasal, Area 2 inferior, Area 2 temporal and Area 3 superior, Area 3 nasal, Area 3 inferior, Area 3 temporal) compared to control eyes.

Considering the same areas and ETDRS sectors, the percentage of abnormal values observed in our MSnoON cohort was reduced with respect to the percentage of abnormal values previously reported in MSON eyes [14]. In fact, in at least five MSnoON eyes, we observed a normal morphological condition, whereas a greater number of MSON eyes showed morphological involvement. Notwithstanding the presence of five MSnoON eyes with normal OCT values, on average, the mean OCT values observed in the MSnoON group were significantly reduced when compared to control ones.

The finding that MSnoON eyes may show mean reduced GCL+ thickness is consistent with our previous observation [8], describing that the sum of all layers composing the inner retina (inner retina macular thickness) was reduced. These data confirm that GCL+ can be considered as the morphological parameter indicative of structural retinal neurodegeneration in MS disorder [2,3,4,15].

Only one other study [32] evaluated the inner retina involvement in MSnoON eyes using an OCT analysis similar to the analysis performed in this study. In fact, Hu et al. [32] observed, in MSnoON eyes, a significant reduction in GC-IPL located in annular areas and ETDRS sectors, particularly in an elliptic island on the nasal side of the fovea. This result was attributable to an impairment in macular smaller axons belonging to the papillo-macular bundle, similarly to optic neuropathies of mitochondrial origins [33,34]. By contrast, in our study, we did not find a similar localized thickness reduction in Area 2 nasal sectors, since the mean GCL+-T was reduced in all studied sectors (see Table 1). The disagreement with the findings by Hu et al. [32] can be ascribed to the non-comparable tool used for macular segmentation analysis and to the intrinsic differences in the study cohort.

The results of our present and previous studies [8,14,32] suggest that, in MS disorder, a primary morphological involvement of inner retina and specifically of macular RGCs, independently from an inflammatory event of the optic nerve, may occur in a large percentage of eyes. It is likely that in the presence of optic neuritis, the pre-existing structural vulnerability of RGCs is exacerbated with a greater morphological involvement detectable in MSON eyes.

### 4.2. Functional Findings

The mfPhNR RAD was considered as the functional parameter reflecting the localized bioelectrical activity of the RGCs and their fibers [14,35].

In the present cohort of MSnoON eyes, we observed a reduction in mfPhNR RADs in a range from 75.00% to 83.3% of eyes considering both rings and ETDRS sectors. Differently from the MSON cohort examined in our previous study [14], we found that a localized RGC dysfunction was present in a greater percentage, ranging from 89.47% to 100.00%. In addition, when considering individual values, only three out of five MSnoON eyes showed normal mfPhNR RAD values, whereas a greater number of eyes presented a significant RGC dysfunction in MSON eyes [14]. The significant mean reduction in mfPhNR RAD in all analyzed rings and sectors found in the MSnoON group, as compared to controls, is indicative of a wide RGC dysfunctional phenomenon involving both more central (macular) and peripheral macular areas.

Indirectly, different electrofunctional methodologies, such as steady-state macular Focal-ERG and 2P component of PERG [12], and full-field photopic negative response [16], investigated previously on the inner retina function of the whole macula, do not selectively isolate the responses of specific macular regions (rings and sectors). In agreement with our results, significant dysfunction of macular RGCs was found in different cohorts of MSnoON eyes [12,16,35]. These previous findings were ascribed to a direct inner retinal involvement or retrograde degeneration due to MS.

In addition, partially consistent with our results, Al-Nosairy et al. [4], by using a multifocal PERG technique, found in MSnoON eyes only an amplitude reduction in the foveal region, whereas in MSON eyes, the inner retina dysfunction was extended to more peripheral retinal areas.

Taken together, the present data describe localized mfPhNR RAD reduction in MSnoON eyes that underlies the more pronounced RGC dysfunction found in MSON eyes [14]. This reiterates the concept that the occurrence of an inflammatory event at the optic nerve level, secondary to MS, significantly and additionally may impair the originally primary dysfunctional macular RGCs.

The contribution of the outer retinal elements’ signals in the genesis of the inner retinal responses can also be considered. However, we [26] and others [36] described normal multifocal ERG RADs in MSnoON eyes, thus assessing the functional integrity of the outer retina (photoreceptors and bipolar cells) in MS eyes in the absence of ON. Therefore, the macular inner retina dysfunction is not influenced by the functional activity of the outer macular elements.

### 4.3. Morpho-Functional Relationships and Correlations

As described in Table 2, the majority of MSnoON eyes (ranging from 58.33% to 79.17%) showed a reduction in both CGL+-T and mfPhNR RAD, indicating that in MSnoON eyes, structural involvement of RGCs might induce inner macular dysfunction, or vice versa.

Nevertheless, it is likely that the functional changes might appear in absence of structural changes. This is derived from the evidence that in the MSnoOn group, a percentage of eyes (ranging from 4.17% to 20.83%) showed reduced mfPhNR RAD with normal GCL+-T. This was observed, in particular, when mfPhNR R1 RADs were compared to GCL+-T Area 1, leading to the observation that the foveal area is functionally more vulnerable than the other areas or sectors. Moreover, all this is supported by the data that in of our MSnoON eyes, reduced GCL+-T and normal mfPhNR RAD were not found.

In the MSnoON cohort, the significant linear correlation between CGL+-T and mfPhNR RAD values described simultaneous morpho-functional RGC damage for central and peripheral macular areas (rings, as in Figure 2, and ETDRS sectors, as in Figure 3 and Figure 4).

These results indicate that in MS, even in absence of ON, the macular RGC degeneration is quite evident at the structural and functional levels and, significantly, involves the whole macula, as derived by the RGC structural damage correlated with the RGC dysfunction. Although a morpho-functional dissociation was observed in some MSnoON eyes (see above), the results of linear correlations are consistent with the similar findings found in MSON eyes [14].

## 5. Conclusions

In conclusion, our results suggest that in relapsing–remitting MS patients (EDSS score between 0 and 3) in the absence of optic nerve inflammation, a potential primary morpho-functional involvement of the inner macular elements occurs. This impairment involves all macular areas and sectors.

Thus, the present study offers scientific bases for the primitive morpho-functional impairment of macular RGCs in MSnoON eyes and a possibility to compare the results with a different population of MSON eyes [14], where the retrograde degeneration after an episode of ON could be responsible for the inner retinal damage.

Nevertheless, we acknowledge that the present study has some limitations, such as the characteristics of the MS group under investigation, limited to relapsing–remitting MS patients with a restricted EDSS score. Further studies may help to confirm whether our conclusions may also be applied to different typologies of MS disease and to different stages of the disease (EDSS score > 3).

## Figures and Tables

**Table 1:** ANOVA analysis of ganglion cell layer + thickness and multifocal photopic negative response amplitude density values from control and multiple sclerosis eyes without a history of optic neuritis.

	Controls (N ^c^ = 30)		MSnoON ^a^ (N^c^ = 24)		ANOVA ^b^	
	Mean	SD ^d^	Mean	SD ^d^	f (1.53)	*p*
**Area 1 GCL+-T ^e^ (µ ^f^)**	48.467	8.080	40.667	7.800	12.81	<0.001
**Area 2 GCL+-T ^e^ (µ ^f^)**	93.408	5.484	83.481	9.866	21.96	<0.001
**Area 3 GCL+-T ^e^ (µ ^f^)**	65.375	4.504	59.479	6.315	16.01	<0.001
**ETDRS ^g^ Area 2 sup ^h^ GCL+-T ^e^ (µ ^f^)**	95.400	5.164	81.000	14.440	25.82	<0.001
**ETDRS ^g^ Area 2 nas ^i^ GCL+-T ^e^ (µ ^f^)**	93.767	6.426	83.292	8.971	24.96	<0.001
**ETDRS ^g^ Area 2 inf ^j^ GCL+-T ^e^ (µ ^f^)**	94.767	6.377	77.875	10.494	53.29	<0.001
**ETDRS ^g^ Area 2 temp ^k^ GCL+-T ^e^ (µ ^f^)**	89.700	6.052	80.458	8.272	22.47	<0.001
**ETDRS ^g^ Area 3 sup ^h^ GCL+-T ^e^ (µ ^f^)**	63.133	4.939	58.667	5.530	9.80	=0.003
**ETDRS ^g^ Area 3 nas ^i^ GCL+-T ^e^ (µ ^f^)**	68.833	4.624	60.375	7.529	20.05	<0.001
**ETDRS ^g^ Area 3 inf ^j^ GCL+-T ^e^ (µ ^f^)**	61.833	4.227	57.000	8.241	7.79	0.007
**ETDRS ^g^ Area 3 temp ^k^ GCL+-T ^e^ (µ ^f^)**	67.700	5.633	57.625	5.106	46.31	<0.001
**R1 ^l^ mfPhNR ^m^ RAD ^n^ (nV/deg^2^) ^o^**	26.274	7.267	17.346	5.295	25.29	0.001
**R2 ^p^ mfPhNR ^m^ RAD ^n^ (nV/deg^2^) ^o^**	11.404	4.163	8.567	2.913	8.00	0.007
**R3 ^q^ mfPhNR ^m^ RAD ^n^ (nV/deg^2^) ^o^**	8.163	2.346	4.978	1.917	28.81	<0.001
**R4 ^r^ mfPhNR ^m^ RAD ^n^ (nV/deg^2^) ^o^**	4.323	1.082	2.865	0.854	29.06	<0.001
**R5 ^s^ mfPhNR ^m^ RAD ^n^ (nV/deg^2^) ^o^**	3.728	0.873	2.313	1.022	30.10	<0.001
**ETDRS ^g^ R2 ^p^ sup ^h^ mfPhNR ^m^ RAD ^n^ (nV/deg^2^) ^o^**	14.437	4.885	10.821	2.268	11.19	=0.002
**ETDRS ^g^ R2 ^p^ nas ^i^ mfPhNR ^m^ RAD ^n^ (nV/deg^2^) ^o^**	16.653	7.191	9.721	3.814	18.16	<0.001
**ETDRS ^g^ R2 ^p^ inf ^j^ mfPhNR ^m^ RAD ^n^ (nV/deg^2^) ^o^**	13.447	6.525	9.367	3.874	7.31	0.009
**ETDRS ^g^ R2 ^p^ temp ^k^ mfPhNR ^m^ RAD ^n^ (nV/deg^2^) ^o^**	17.520	5.210	7.988	2.997	63.39	<0.001
**ETDRS ^g^ R3 + R4 ^t^ sup ^h^ mfPhNR ^m^ RAD ^n^ (nV/deg^2^) ^o^**	5.983	1.906	4.550	1.905	7.54	=0.008
**ETDRS ^g^ R3 + R4 ^t^ nas ^i^ mfPhNR ^m^ RAD ^n^ (nV/deg^2^) ^o^**	5.513	1.626	3.967	1.299	14.35	<0.001
**ETDRS ^g^ R3 + R4 ^t^ inf ^j^ mfPhNR ^m^ RAD ^n^ (nV/deg^2^) ^o^**	5.710	1.705	4.513	1.558	7.09	=0.010
**ETDRS ^g^ R3 + R4 ^t^ temp ^k^ mfPhNR ^m^ RAD ^n^ (nV/deg^2^) ^o^**	7.287	2.111	4.133	1.209	42.35	<0.001

^a^ MSnoON = multiple sclerosis eyes without a history of optic neuritis; ^b^ ANOVA = one-way analysis of variance. *p*-value < 0.01 was considered as statistically significant for group comparisons; ^c^ N = number of eyes; ^d^ SD = 1 standard deviation; ^e^ GCL+-T = ganglion cell layers complex thickness; ^f^ µ = micron; ^g^ ETDRS = Early Treatment Diabetic Retinopathy Study map configuration; ^h^ sup = superior sector; ^i^ nas = nasal sector; ^j^ inf = inferior sector; ^k^ temp = temporal sector; ^l^ R1 = concentric annular areas (rings) analyzing 5° radius circular area centered on the fovea; ^m^ mfPhNR = multifocal photopic negative responses; ^n^ RAD = response amplitude density; ^o^ nV/deg^2^ = nanoV/degree^2^; ^p^ R2 = ring within 5° and 10°; ^q^ R3 = ring within 10° and 15°; ^r^ R4 = ring within 15° and 20°; ^s^ R5 = ring within 20° and 25°; ^t^ R3 + R4 = annular area between 10° and 20°.

**Table 2:** Normal or reduced values of multifocal photopic negative response amplitude densities and ganglion cell layer + thickness observed in multiple sclerosis eyes without a history of optic neuritis for correspondent macular areas/rings and sectors.

	Normal mfPhNR ^a^ and GCL+ ^b^-T ^c^ Number of Eyes and (%) ^d^	Reduced mfPhNR ^a^ and GCL+ ^b^-T ^c^ Number of Eyes and (%) ^d^	Normal mfPhNR ^a^ and Reduced GCL+ ^b^-T ^c^ Number of Eyes and (%) ^d^	Reduced mfPhNR ^a^ and Normal GCL+ ^b^-T ^c^ Number of Eyes and (%) ^d^
**mfPhNR ^a^ R1 ^e^ vs. GCL+ ^b^-T ^c^ Area 1**	4 (16.67)	15 (62.50)	0 (0.00)	5 (20.83)
**mfPhNR ^a^ R2 ^e^ vs. GCL+ ^b^-T ^c^ Area 2**	5 (20.83)	19 (79.17)	0 (0.00)	0 (0.00)
**mfPhNR ^a^ R3 + R4 ^e^ vs. GCL+ ^b^-T ^c^ Area 3**	5 (20.83)	17 (70.83)	0 (0.00)	2 (8.33)
**mfPhNR ^a^ ETDRS ^f^ R2 ^e^ Suprior vs. GCL+ ^b^-T ^c^ ETDRS ^f^ Area 2 Superior**	5 (20.83)	18 (75.00)	0 (0.00)	1 (4.17)
**mfPhNR ^a^ ETDRS ^f^ R2 ^e^ Nasal vs. GCL+ ^b^-T ^c^ ETDRS ^f^ Area 2 Nasal**	5 (20.83)	18 (75.00)	0 (0.00)	1 (4.17)
**mfPhNR ^a^ ETDRS ^f^ R2 ^e^ Inferior vs. GCL+ ^b^-T ^c^ ETDRS ^f^ Area 2 Inferior**	5 (20.83)	19 (79.17)	0 (0.00)	0 (0.00)
**mfPhNR ^a^ ETDRS ^f^ R2 ^e^ Temporal vs. GCL+ ^b^-T ^c^ ETDRS ^f^ Area 2 Temporal**	3 (12.50)	20 (83.33)	0 (0.00)	1 (4.17)
**mfPhNR ^a^ ETDRS ^f^ R3+ R4 ^e^ Suprior vs. GCL+ ^b^-T ^c^ ETDRS ^f^ Area 3 Superior**	5 (20.83)	14 (58.33)	0 (0.00)	5 (20.83)
**mfPhNR ^a^ ETDRS ^f^ R3+ R4 ^e^ Nasal vs. GCL+ ^b^-T ^c^ ETDRS ^f^ Area 3 Nasal**	4 (16.67)	16 (66.67)	0 (0.00)	4 (16.67)
**mfPhNR ^a^ ETDRS ^f^ R3+ R4 ^e^ Inferior vs. GCL+ ^b^-T ^c^ ETDRS ^f^ Area 3 Inferior**	4 (16.67)	19 (79.17)	0 (0.00)	1 (4.17)
**mfPhNR ^a^ ETDRS ^f^ R3 + R4 ^e^ Temporal vs. GCL+ ^b^-T ^c^ ETDRS ^f^ Area 3 Temporal**	5 (20.83)	15 (62.50)	0 (0.00)	4 (16.67)

^a^ mfPhNR = multifocal photopic negative responses; ^b^ GCL + = ganglion cell layers complex thickness; ^c^ T = Thickness; ^d^ (%) = percentage; ^e^ R1, R2, R3, R4 = concentric Rings centered on the fovea, R1 refers to 5° radius circular area, R2 refers to the annulus between 5° and 10°, R3 refers to the annulus between 10° and 15° and R4 refers to the annulus between 15° and 20°; ^f^ ETDRS = Early Treatment Diabetic Retinopathy Study map configuration. In MSnoON eyes, when the individual morphological (GCL+-T) data were plotted against the functional (mfPhNR RAD) data, statistically significant (*p* < 0.01) linear correlations in both Rings and ETDRS sectors were observed.

## Data Availability

Original data are available on request.

## Supplementary Materials

The following supporting information can be downloaded at https://www.mdpi.com/article/10.3390/jcm14165919/s1: Table S1: Individual ganglion cell layer + thickness and multifocal photopic negative response amplitude densities data observed in multiple sclerosis eyes without history of optic neuritis.

## Abbreviations

MS	multiple sclerosis
RGCs	retinal ganglion cells
ON	optic neuritis
OCT	optical coherence tomography
GCIPL	ganglion cell/inner plexiform layer
MSON	multiple sclerosis patients with optic neuritis
PERG	pattern electroretinogram
mfPhNR	multifocal Photopic Negative Response
ETDRS	Early Treatment Diabetic Retinopathy Study
MSnoON	multiple sclerosis patients without history of optic neuritis
RNFL	retinal nerve fiber layer
ff-PhNR	full-field Photopic Negative Response
SS-OCT	swept-source optical coherence tomography
GCL+-T	ganglion cells layer thickness
IPL	inner plexiform layer
INL	inner nuclear layer
RAD	response amplitude density
R	Ring
sup	superior sector
nas	nasal sector
inf	inferior sector
temp	temporal sector
BCVA	best corrected visual acuity
SD	one standard deviation of the mean
N	number of eyes of each group
ANOVA	one-way analysis of variance
ISCEV	International Society for Clinical Electrophysiology of Vision
