# Supplementary material for: Structural and Functional Assessment of the Macular Inner Retinal Layers in Multiple Sclerosis Eyes Without History of Optic Neuropathy
Source: J Clin Med. 2025 Aug 21;14(16):5919. doi: 10.3390/jcm14165919 (PMC12387542; doi:10.3390/jcm14165919)
Supplement: Supplementary file 1 [file jcm-14-05919-s001.zip › jcm-3736693-supplementary.pdf]

Supplementary Materials

Table S1. Individual Ganglion Cell Layer + Thickness and multifocal Photopic Negative Response Amplitude Densities data observed in Multiple Sclerosis eyes without history of Optic Neuritis

	**Rings/Areas**						**ETDRS [a] Area 2**								**ETDRS [a] Area 3**							
	**R1 [d] /Area 1**		**R2 [d] /Area 2**		**R3+4 [d] /Area 3**		**SUPERIOR**		**NASAL**		**TEMPORAL**		**INFERIOR**		**SUPERIOR**		**NASAL**		**TEMPORAL**		**INFERIOR**	
	**PhNR [b] R1 [d] RAD [e] (nV/ deg^2) [g]**	**GCL+ [c] T [f] (μ) [h]**	**PhNR [b] R2 [d] RAD [e] (nV/ deg^2) [g]**	**GCL+ [c] T [f] (μ) [h]**	**PhNR [b] R3 [d] RAD [e] (nV/ deg^2) [g]**	**GCL+ [c] T [f] (μ) [h]**	**PhNR [b] R2 [d] RAD [e] (nV/ deg^2) [g]**	**GCL+ [c] T [f] (μ) [h]**	**PhNR [b] R2 [d] RAD [e] (nV/ deg^2) [g]**	**GCL+ [c] T [f] (μ) [h]**	**PhNR [b] R2 [d] RAD [e] (nV/ deg^2) [g]**	**GCL+ [c] T [f] (μ) [h]**	**PhNR [b] R2 [d] RAD [e] (nV/ deg^2) [g]**	**GCL+ [c] T [f] (μ) [h]**	**PhNR [b] R3+R4 [d] RAD [e] (nV /deg^2) [g]**	**GCL+ [c] T [f] (μ) [h]**	**PhNR [b] R3+R4 [d] RAD [e] (nV/ deg^2) [g]**	**GCL+ [c] T [f] (μ) [h]**	**PhNR [b] R3+R4 [d] RAD [e] (nV/ deg^2) [g]**	**GCL+ [c] T [f] (μ) [h]**	**PhNR [b] R3+R4 [d] RAD [e] (nV/ deg^2) [g]**	**GCL+ [c] T [f] (μ) [h]**
#1	16.2	35	7.5	97.25	8.4	68.4	8.9	102	12.2	92	11.2	99	13.0	96	5.8	62	6.8	68	7.2	75	6.2	61
#2	9.6	50	3.2	75.25	9	66.75	10.9	90	8.1	91	15.0	91	8.1	86	8.7	66	6.6	72	7.4	69	6.1	62
#3	29.4	46	12.1	89.5	5.8	61.25	12.3	80	4.3	78	11.7	83	8.7	80	3.6	62	2.8	60	2.6	55	4.2	59
#4	17	37	7.3	67	2.7	59.5	6.4	79	4.7	79	6.9	79	7.4	73	4.4	56	3.4	60	4.1	57	4.9	65
#5	11.2	27	9.6	94.6	2.6	61.57	9.3	87	8.5	87	5.0	82	7.1	80	1.8	47	2.8	68	4	57	5.3	63
#6	24.8	56	12.2	80.75	2.4	58.25	9.1	82	9.8	83	9.5	84	4.4	80	2.7	56	4.5	59	4.6	55	3.2	63
#7	12.4	43	6.3	73.25	4.8	58	9.3	77	12.3	63	16.0	65	2.0	69	5.2	60	4.2	56	4.8	54	4.6	62
#8	14.9	40	6.2	80.25	1.5	56	15.7	87	13.3	93	8.1	90	7.0	89	4.5	54	4.7	55	5.2	52	3.4	59
#9	21.8	42	5.7	82.75	6.4	65.03	8.0	98	7.0	98	8.5	99	9.5	95	5	64	4.7	69	5.9	70	4.4	63
#10	15.1	40	10	85.25	3	56.75	12.9	81	9.9	82	11.0	79	9.0	81	2.9	56	3.1	56	3.5	52	3.5	57
#11	11.6	43	6.4	85.25	2.4	49.75	9.2	74	9.6	70	11.3	71	6.7	78	3.4	52	2.1	48	3	42	2.5	57
#12	20.4	42	10.4	82.25	7.8	66	8.6	93	3.8	91	13.6	95	13.5	89	5.1	65	4.6	67	3.3	63	3.8	56
#13	14.3	48	13.4	92	5.9	62.5	13.7	85	10.6	84	6.7	82	3.4	83	4	62	2.5	60	6	59	2.9	56
#14	17.1	45	9.5	82.5	3.7	57	11.2	82	7.7	80	12.0	81	7.8	77	3.9	57	3.7	56	5.3	54	3.5	55
#15	10.8	47	2.7	65	5.2	63	8.9	87	10.5	82	6.2	87	7.4	85	4.3	60	4.6	61	1.8	48	3	54
#16	12.3	37	7.8	73.25	7.1	65.5	12.3	86	12.0	87	6.6	86	6.4	82	5.8	65	4.3	68	3.6	58	3.2	53
#17	22	41	10.7	94.4	8.5	67.25	10.2	84	11.0	80	9.2	80	10.6	86	9.6	64	5.3	69	7.1	66	6.7	64
#17	23.9	50	5.9	73.75	3.1	51	7.6	74	11.3	86	12.3	70	8.2	71	2.9	49	4.5	54	3.2	48	4.2	53
#19	18.4	30	11.2	98.6	2.2	54.5	15.2	64	23.0	62	12.0	63	9.0	64	2.5	54	4.1	50	4.8	54	3.8	52
#20	13.6	37	5.8	83.5	3.6	54	7.7	73	13.2	72	14.0	73	7.7	75	3.5	53	3.5	60	2.7	51	4.6	52

#21	13.3	28	9	81.4	3.2	63.5	12.1	89	10.1	88	3.5	85	2.6	81	3.8	63	2.8	64	2.7	58	2.9	51
#22	22	39	9	77.5	4..3	46.5	12.9	66	6.9	66	8.9	67	8.9	69	4.3	55	2.2	42	4.2	44	2.4	51
#23	23.9	49	10.2	89.75	7.4	66.25	9.5	84	4.6	83	5.8	85	8.0	79	7.8	67	5.3	68	5.9	68	5.9	66
#24	20.3	49	14.5	97.5	2.7	49.25	13.0	47	14.0	46	5.9	46	7.7	58	3.7	59	2.1	59	5.4	59	4	49
CL [i]	23.43	45.62	10.46	91.36	7.36	63.69	12.61	93.47	13.96	91.36	15.57	87.44	11.01	92.38	5.27	61.28	4.91	67.10	6.49	65.59	5.07	60.25

[a] ETDRS= Early Treatment Diabetic Retinopathy Study map configuration; [b] PhNR = multifocal photopic negative responses; [c] GCL + T = ganglion cell layers complex thickness; [d] R1, R2, R3, R4 = concentric annular areas (Rings) centered on the fovea, R1 refers to 5° radius circular area, R2 refers to annular area enclosed between 5° and 10° centered on the fovea, R3 refers to annular area enclosed between 10° and 15° centered on the fovea and R4 refers to annular area enclosed between 15° and 20° centered on the fovea; [e] RAD= response amplitude density; [f] T= Thickness; [g] nV/deg^2 = nanoV/degree^2; [h] μ = micron; [i] CL = 95% Lower Confidence limit derived from Controls
